# Anatomical and Functional Outcomes of Bladder Exstrophy Patients – A Single-Centre Indonesian Experience

**DOI:** 10.5704/MOJ.2511.011

**Published:** 2025-11

**Authors:** A Kurniawan, H Jonathan, R Wikanjaya, LP Aryandhani

**Affiliations:** 1 Department of Orthopaedics and Traumatology, Dr. Cipto Mangunkusumo Hospital, Jakarta, Indonesia; 2 Department of Orthopaedics and Traumatology, Universitas Indonesia, Jakarta, Indonesia; 3 Department of Orthopaedics, Rumah Sakit Khusus Bedah Rawamangun, Jakarta, Indonesia

**Keywords:** bladder exstrophy, pubic diastasis, PedsQL, pelvic osteotomy

## Abstract

**Introduction:**

Bladder exstrophy is an embryologic malformation characterised by a deficiency of the anterior midline, resulting in urogenital and skeletal complications. The aim of this article is to identify the factors influencing outcomes in patients with bladder exstrophy, a condition for which published literature remains limited.

**Material and Methods:**

A retrospective cohort study analysed medical records from 2011 to 2017. Patients with bladder exstrophy who underwent anterior and posterior innominate osteotomy were evaluated for clinical outcomes in an orthopaedic outpatient clinic. Anatomical outcomes were assessed by calculating the percentage of pubic approximation, while functional outcomes were measured using the PedsQL 4.0 and compared to typical peers.

**Results:**

Nineteen children (median age 6 years) were included: 11 boys (57.9%) and 8 girls (42.1%). Among them, 17 had bladder exstrophy, 2 had cloacal exstrophy, 2 had epispadias, and 1 had hypospadias. Sixteen children (84.2%) had no other congenital anomalies. The median age at surgery was 6 months (range 1–71 months), with a median pubic approximation percentage of 78.5% (range 65– 98.1%). The mean PedsQL score post-operation was 97.2 ± 1.6. A significant correlation was found between age at operation, post-operative diastasis, pubic approximation percentage, and PedsQL score (p < 0.05).

**Conclusion:**

The clinical outcomes for bladder exstrophy patients were favourable, as indicated by pubic approximation percentage and PedsQL scores. Age at operation and post-operative diastasis significantly influenced both anatomical and functional outcomes.

## Introduction

Bladder exstrophy is a congenital abnormality characterised by an open anterior abdominal wall and exposed bladder, representing a complex congenital condition with impacts on the urinary and musculoskeletal systems. Cloacal exstrophy involves these systems and additionally affects the digestive tract, attributed to developmental disruptions of the cloacal membrane during early embryogenesis. This condition may coexist with epispadias or be part of a complex involving other congenital abnormalities such as omphalocele, anal atresia, and spinal defects. The occurrence of bladder exstrophy is around 1 in 30 000 to 50 000 live births, with a variable gender ratio, while cloacal exstrophy is rarer, prompting considerations that include fatalities in utero in incidence rates^1,2^. The management of bladder exstrophy today requires a multidisciplinary approach, focusing not only on survival but also on optimising patient function, psychosocial development, and overall quality of life, with interventions like pelvic osteotomy to correct musculoskeletal deformities^3-6^.

However, research on the long-term outcomes of bladder exstrophy, including anatomical and functional aspects, remains limited. Studies from Kouame *et al* and Gupta *et al* reported positive quality of life and moderate continence function over periods up to 20 years post-treatment, though searches on PubMed reveal relatively few results compared to other conditions. This underscores the need for comprehensive long-term studies, particularly in Indonesia, where bladder exstrophy surgeries have been performed since 2011 at our centre^3,4^.

This study aims to evaluate the anatomical outcomes and quality of life among bladder exstrophy patients, utilising the Paediatric Quality of Life Inventory 4.0 (PedsQL 4.0) Generic Core questionnaire^7^ to assess health-related quality of life across physical, emotional, social, and educational domains. The questionnaire is a validated tool for paediatric patients, designed to measure health risk, monitor health status, and evaluate therapeutic outcomes. By providing a long-term view of patient conditions post-operation, this research contributes to the understanding of bladder exstrophy treatments in Indonesia and aims to inform national healthcare services to enhance safety and quality of life for patients with this serious congenital disease.

## Materials and Methods

This study is analytic descriptive research utilising a retrospective cohort design. There are two dependent variables in this study, which include anatomical outcomes represented by the percentage of pubic approximation and functional outcomes assessed through the Indonesian version PedsQL 4.0 Generic Core questionnaire published by Varni *et al*^7^. Questionnaire translation and validation was performed officially by The Mapi Research Trust^8,9^. The independent variables analysed in this study include current age, age at the time of surgery, post-operative period, gender, type of intervention, pre-operative pubic diastasis, and postoperative pubic diastasis.

The subjects of this research were patients with bladder exstrophy who attended our centre between the years 2011 and 2017 and underwent anterior or posterior innominate osteotomy surgery. We perform posterior innominate osteotomy in patients aged one month or younger, as they have more flexible pelvises, which facilitates pubic closure. Otherwise, we perform anterior innominate osteotomy. We also added additional symphysis pubis plating in patients older than six months. A total sampling technique was employed in this study due to the rarity of bladder exstrophy cases. The exclusion criteria applied included patients who passed away before treatment, patients who died after the procedure, patients who refused therapy, patients who declined to participate in the study, and patients who could not be contacted again.

**Fig. 1:**
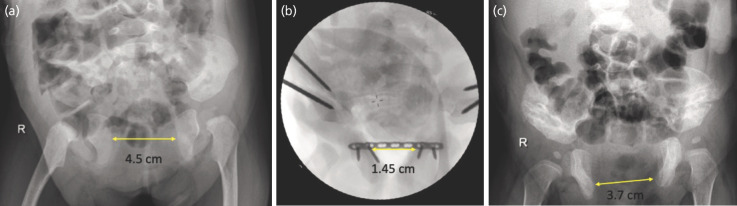
Diastasis measurement of patient (a) pre-operatively, (b) post-operatively, (c) final follow-up.

Data collection is conducted through the examination of patients' symphysis diastasis. Bladder exstrophy patients underwent plain pelvic radiographs with an anteroposterior projection, taken before and after the surgery. From the radiographic results, diastasis was measured as the distance between the two most medial points of the pubic ramus (Fig. 1). Along with pre-operative results, these values are used to calculate the approximation of pubic diastasis. The patient’s parents are also requested to fill out the PedsQL questionnaire according to the patient's age at the time: 2–4, 5–7, 8–12, and 13–18 years, with scores ranging from 0 to 100, where 100 reflects the best quality of life.

The parameters presented in the study analysis were the patient's age, age at surgery, gender, type of bladder exstrophy, and values of pre-operative diastasis, postoperative diastasis, pubic approximation percentage, preoperative PedsQL score, and post-operative PedsQL score. The gathered data samples were analysed using the statistical computer software SPSS (Statistic Program for Social Science) version 20. The analysis of the relationship between variables such as current age, age at surgery, diastasis before and after surgery in relation to approximation presentation and PedsQL scores utilised Pearson's correlation test for normally distributed data and Spearman's correlation test for data that were not normally distributed. Meanwhile, the analysis of the relationship between variables such as postoperative period, gender, type of procedure in relation to approximation presentation and PedsQL scores employed the independent t-test for normally distributed data and the Mann-Whitney test for data that were not normally distributed.

The implementation of this research adheres to the principles of the "Helsinki Declaration" and guidelines outlined in the "Guideline for Good Clinical Practice" from the ICH Tripartite Guideline. Ethical clearance for this research was granted from the Committee on Medical Research Ethics of the Faculty of Medicine, University of Indonesia No. 763/UN.F1/ETIK/2017.

## Results

**Fig. 2:**
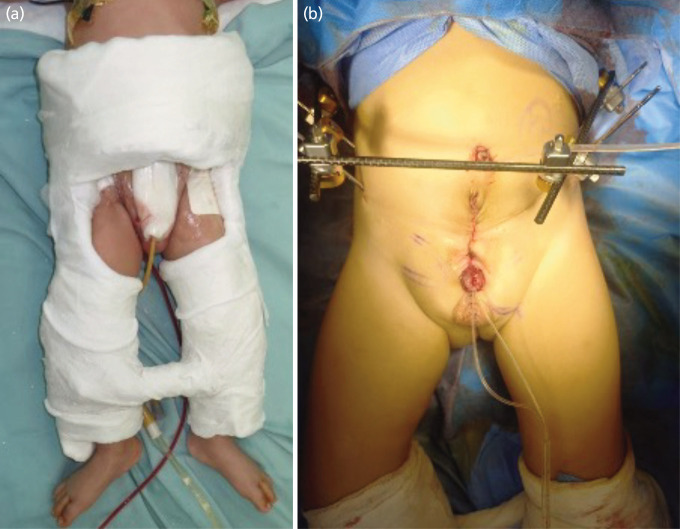
Bladder exstrophy fixation using (a) hip spica cast, (b) external fixation.

The subjects’ characteristic of this study can be seen in Table I and II. Total subject of this study was 19 with the majority of subjects are male (57.9%) with a mean age of 4.7 years with a range of 1.5–9 years old. The anomaly type is predominantly bladder exstrophy in 17 cases (89.5%) compared to cloacal exstrophy with related organ anomalies include 2 cases of epispadias (10.5%), 1 case of hypospadias (5.3%). We perform fixation using casting in 10 cases (52.6%) and external fixation in 9 cases (47.4%) and added additional symphysis pubis plating in 10 patients (52.6%) (Fig. 2). We previously routinely performed casting for postoperative fixation; however, using a hip spica cast made it difficult to evaluate bowel function and can also even increase abdominal pressure causing bowel problem such as cast syndrome^10^. Subsequently, we transitioned to routinely performing external fixation after surgery. We currently prefer external fixation as it is easier for surgical wound care, skin hygiene, and evaluation bowel function. Postoperatively there was 2 cases (10.5%) of skin irritation related to casting and 2 case of pin tract infection related to external fixation application. However, there were no major complications requiring further surgery.

**Table I:** Measurement of parameters based on gender.

Parameters	Total					
	Male		Female		Total	
	(N = 19) Mean ± SD / Median (QR)	(N = 11) Range	(N = 8) Mean ± SD / Median (QR)	Range	Mean ± SD / Median (QR)	Range
Age (year)	4.7 ± 2.6	1.5 - 9	5.1 ± 2.1	1 - 8	4.8 ± 2.4	1 - 9
Age at operation (month)	6 (9)	1 - 71	9 (23)	1 - 48	6 (21)	1 - 71
Pre-operative diastasis (cm)	6.7 (0.8)	5.2 - 8.1	6.2 (1.2)	5.5 - 7.7	6.4 (0.6)	5.1 - 8.1
Post-operative diastasis (cm)	2.2 (0.2)	1.1 - 3.2	2.2 (1.0)	1.1 - 2.4	2.2 (0.4)	1.1 - 3.2
Median PedsQL score post-op	96.8	94.6 - 100	97.5	95.1 - 100	96.7	94.6 - 100

**Table II:** Subjects characteristic based on type of bladder exstrophy, related organ anomalies, post-operative period, and fixation method.

Variable	Total (N = 19)	
	n	%
Type of Bladder Exstrophy		
Bladder	17	89.5
Cloaca	2	10.5
Related Organ Anomalies		
Epispadia	2	10.5
Hypospadia	1	5.3
None	16	84.2
Innominate osteotomy		
Anterior	13	68.4
Posterior	6	31.6
Fixation Method		
External Fixation	9	47.4
Casting	10	52.6
Post-operative Period		
≤ 36 months	10	52.6
> 36 months	9	47.4
Complication		
Skin irritation	2	10.5
Pin tract infection	2	10.5
Symphysis pubis plating		
Yes	10	52.6
No	9	47.4

**Fig. 3:**
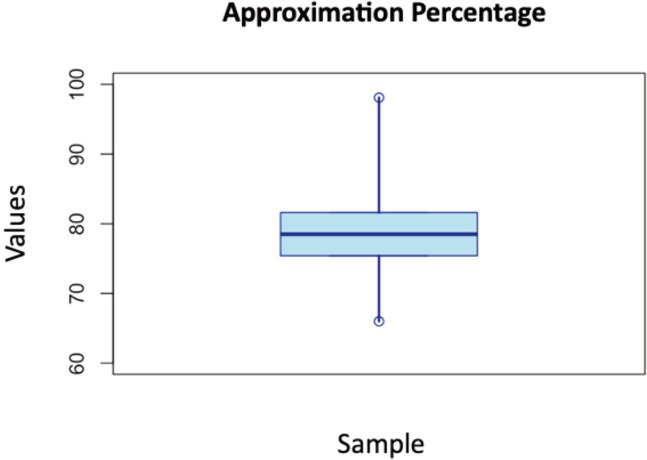
Boxplot of approximation percentage.

Among the subjects, the median pre-operative diastasis was found to be 6.4cm with a range of 5.1–8.1cm, while the postoperative diastasis median was 2.2cm with a range of 1.1–3.2. Based on the gender distribution, both pre-operative and post-operative diastasis was found to be higher in male subjects. Meanwhile, post-operative mean PedsQL score was found to be 97.2 ± 1.6 with a range of 94.6–100 and higher in female subjects. The calculation of the pubic approximation percentage showed a median value of 78.5% with a range of 65.0–98.1 (Fig. 3).

This research discovers the correlation between age and anatomical outcome which was measured with diastasis before surgery, after surgery, and the percentage of approximation. The results indicated that there was no significant correlation between age and anatomical outcomes, including diastasis before surgery, diastasis after surgery, and the percentage of approximation (p>0.05). However, the analysis of the relationship between age at the time of surgery and anatomical outcome values reveals a significant correlation between age and post-operative diastasis (p=0.043) as well as the percentage of approximation (p=0.009) (Table III).

**Table III:** Correlation between age and anatomical outcomes.

Variable_1_	Variable_12_	Correlation-r	p-value
Age*	Pre-operative diastasis	-0.221	0.364
	Post-operative diastasis	-0.246	0.309
	Percentage of approximation	0.020	0.939
Age at surgery*	Pre-operative diastasis	-0.429	0.067
	Post-operative diastasis	0.469	0.043
	Percentage of approximation	-0.770	0.009

Note - *Analysed using Spearman’s non-parametric test

The relationships between gender, fixation method, diastasis approximation percentage and various outcome measures including PedsQL post-operative and post-operative diastasis were also evaluated. As we can see on the Table IV, all variables show no statistically significant difference.

**Table IV:** Relationships between parameters and percentage of approximation, PedsQL post-operative, and post-operative diastasis.

Subject Characteristics			Total (N = 19)			
	Percentage of approximation		PedsQL post-operative		Post-operative diastasis	
	Median (QR) / Mean ± SD	p-value	Median (QR) / Mean ± SD	p-value	Median (QR) / Mean ± SD	p-value
Gender						
Male	78.5 (3.7)	0.904^a^	97.0 ± 1.5	0.638^a^		
Female	78.1 (18.2)		97.4 ± 1.7			
Fixation method						
External fixation	79.1 ± 8.5	0.672^b^	97.3 ± 1.5	0.977^b^	4.0 ± 0.8	0.530^b^
Casting	80.9 ± 9.7		97.2 ± 1.8		4.7 ± 0.6	

Notes - ^a^ : The p-value was calculated using the Mann-Whitney non-parametric test, ^b^ : The p-value was calculated using the unpaired T parametric test

Age and post-operative PedsQL scores showed no significant relationship (p=0.637, r=0.116). Regarding the variable of age at the time of surgery, the results demonstrate a significant relationship between surgery age and postoperative PedsQL scores (p=0.001), emotional functioning (p=0.016), and school functioning (p=0.009) (Table V).

**Table V:** Relationship between age and post-operative PedsQL scores.

Variable1	Variable12	Correlation-r	p-value*
Age	Mean score post-op PedsQL	0.116	0.637
	Physical function	0.189	0.439
	Emotional function	0.058	0.813
	Social function	-0.271	0.263
	Intelligence function	0.165	0.501
Age at surgery	Mean score post-op PedsQL	-0.761	0.001**
	Physical function	-0.378	0.111
	Emotional function	-0.545	0.016**
	Social function	-0.343	0.151
	Intelligence function	-0.584	0.009**

Notes - * : The p-value was calculated using the Spearman non-parametric test, ** : Statistically significant (p<0.05)

In relation to the variable of approximation percentage, the results show a significant association between the approximation percentage and post-operative PedsQL scores (p=0.007), emotional functioning (p=0.001), and intelligence functioning (p=0.002). The analysis of this relationship can be seen in Table VI.

**Table VI:** Relationship between diastasis and PedsQL score.

Variable_1_	Variable_12_	Correlation-r	p-value*
Pre-operative diastasis	Mean score post-op PedsQL	0.148	0.546
	Physical function	-0.134	0.585
	Emotional function	0.155	0.528
	Social function	0.160	0.513
	Intelligence function	0.155	0.528
Post-operative diastasis	Mean score post-op PedsQL	-0.695	0.001**
	Physical function	-0.551	0.015**
	Emotional function	-0.559	0.013**
	Social function	0.081	0.741
	Intelligence function	-0.637	0.003**
Approximation percentage	Mean score post-op PedsQL	0.879	0.007**
	Physical function	0.423	0.071
	Emotional function	0.761	0.001**
	Social function	0.140	0.569
	Intelligence function	0.809	0.002**

Notes - * : The p-value was calculated using the Spearman non-parametric test, ** : Statistically significant (p<0.05)

## Discussion

In this study, the median age at the time of surgery for patients with bladder exstrophy was 6 months, with a minimum age of 1 month and a maximum age of 71 months. This was earlier compared to a study by Jones *et al*, where the average age at the time of surgery for patients with bladder exstrophy was 15.5 months. Additionally, the average age of patients with bladder exstrophy at follow-up was 4.8 ± 2.4 years, which was not significantly different from the study conducted by Jones *et al*, where the average patient age was 3 years^11^.

The results of the study indicated no significant correlation between patient age and pre-operative diastasis, postoperative diastasis, and approximation percentage. This is consistent with a study by Jones *et al*, stating that patient age at follow-up does not determine post-operative anatomical outcomes. However, each age range has different values for normal approximation percentages^11^.

This study results indicated a correlation between age at surgery with post-operative diastasis and approximation percentage. This shows that earlier surgical treatment will benefit in achieving a lower post-operative diastasis with a higher percentage of approximation. This is consistent with a study by Baird *et al*, stating that anatomical outcomes of patients with bladder exstrophy are better if surgery is performed early after birth^12^. Study by Jones *et al* also stated that performing osteotomy procedures within the first three years of birth can lead to better closure of the pubic symphysis^11^. Furthermore, according to Castagnetti *et al*, approximation percentage, the most important clinical outcome in assessing patients with bladder exstrophy, is highly influenced by the patient's age at the start of the surgery^13^.

Gender did not have a significant influence on the approximation percentage. This finding aligns with a study by Jones *et al*, indicating that patient age and gender do not affect clinical outcomes of patients with bladder exstrophy post-surgery^11^.

Significant improvement was observed in symphysis pubis diastasis, with a median pre-operative diastasis measurement of 6.4cm and a post-operative median of 2.2cm. This indicates that surgical interventions performed on patients with bladder exstrophy can improve diastasis approximation by a median of 78.5%. This is a significant achievement considering the research by Meldrum *et al*, which stated that osteotomy procedures performed to approximate pubis diastasis can decrease intra-abdominal pressure and strengthen the pelvic base supporting the bladder^14^. In addition, the percentage of diastasis approximation in this study was higher than that in the study by Jones *et al*, where the average percentage of approximation was 37%^11^.

The method of fixation did not show a significant impact on the approximation percentage. This is in agreement with a study by Kurbet *et al*, indicating that the fixation method does not affect post-operative diastasis^15^. Shnorhovarian *et al* also stated that the fixation method does not influence the surgical outcomes. Although Meldrum *et al* stated that external fixation has some advantages over cast placement, a study by Arlen *et al* suggested that fixation with spica is an effective method with low complication rates^16,17^.

In regard to the relationship between the fixation method and the difference in diastasis before and after surgery, the results showed no significant correlation (p>0.05). This aligns with a study by Shnorhovarian *et al*, stating that the fixation method does not affect outcomes post-surgery^16^. Fixation with cast alone has been considered a safe and effective method as shown in a study by Arlen *et al*^17^.

There was no significant relationship between the fixation method and the difference in PedsQL scores before and after surgery. This supports a recent study by Baumgartner *et al*, which indicated that failure of an operation can occurs across all fixation methods. External fixation itself provides comfort for both patients and healthcare providers, as indicated in this study^18^. This supports a prior study by Meldrum *et al*, stating that the risk of skin injuries with cast usage should be considered in selecting the fixation method^14^. A study by Kajbafzadeh *et al* regarding the use of a biodegradable plate fixation system post-bladder exstrophy operation may be a consideration for future fixation methods^19^. Additionally, a study by Inouye *et al* also revealed that the management of bladder exstrophy patients can lead to complications^20^. In our study there was as two of skin irritation and two case of pin tract infection related to the management options used.

Functional parameters were evaluated using PedsQL. The study found an increase in the average PedsQL score before surgery from 37.1 to an average of 96.7 after surgery. This evaluation aligns with the review conducted by Joseph G Borer *et al*, concluding a significant improvement in anatomical, psychological, and social life conditions of patients with bladder exstrophy before and after surgery^21^. Furthermore, there was no significant relationship between patient age and PedsQL scores after surgery. This is in line with the study stating that patient age at follow-up does not determine quality of life, but specific age groups serve as benchmarks or indicators for determining quality of life^21^. In addition, according to Kurbet *et al*^15^, there was no significant association between patient age with bladder exstrophy and PedsQL scores. Consistent with Kurbet's study, a study by Castagnetti *et al*^13^ on functional outcomes of bladder exstrophy patients measured with questionnaire showed no significant relationship between age and clinical outcomes.

There was no significant difference between male and female genders concerning PedsQL scores after surgery. This is in accordance with a study by Kurbet *et al*, stating that gender does not impact the quality of life of patients with bladder exstrophy; rather, it is influenced by parental factors and the patient's environment^15^.

The study results showed a significant relationship between age at surgery and PedsQL scores after surgery. Analysing the age at surgery concerning the PedsQL item after surgery, a significant relationship was found for emotional factors and intelligence. This indicates that the earlier the patient undergoes surgery, the higher the functional outcome, specifically their emotional and intelligence outcome. According to Borer *et al*, patients who undergo osteotomy procedures earlier will have better closure of the pubic symphysis, enhancing motor function, especially related to pelvic movements, which also affects patients' psychosocial function^21^.

The analysis between post-operative diastasis and approximation with functional outcome measured using PedsQL scores after surgery shows a moderate correlation. Meanwhile, pre-operative diastasis does not show no significant correlation. This is in line with a study by Borer *et al*, stating that PedsQL scores are more influenced by the patient's post-operative condition. However, parental factors and the surrounding environment greatly influence the quality of life of patients with bladder exstrophy^21^.

Furthermore, the analyses indicated a significant correlation between approximation percentage and PedsQL item after surgery, such as physical, emotional, and intelligence function. This aligns with a study by Meldrum *et al*, which stated that post-operative anatomical outcomes greatly influence motor function, especially related to pelvic movements, which in turn affects psychosocial function of patients^14^.

There was no significant relationship between the fixation method and type of procedure with PedsQL scores. This is consistent with a study by Kurbet *et al*, stating that there is no significant association between the fixation method and the quality of life of patients with bladder exstrophy^15^. Baumgartner *et al* also concluded that failures in the operation can occur with any fixation method^18^.

The study has several limitations that may affect its findings. This study was performed in single-centre with a small sample size. However, due to the rarity of the condition during our six-year cohort we were only able to obtain 19 patients. A previous study by Borer *et al* with a study period of eight years also only yielded 23 patients^21^. Additionally, due to the retrospective nature of the study potential recall bias may occur when completing the PedsQL questionnaire. Lastly, the lack of long-term follow-up on this study. Despite this, the study gives several insights on functional and anatomical outcome on a rare condition such as bladder exstrophy.

## Conclusion

From this study, it can be concluded that there is clinical improvement in operated patients, as observed from both the approximation presentation and post-operative PedsQL scores. Additionally, there is a relationship between age at operation and diastasis after surgery with functional outcomes of bladder exstrophy patients. Further research is necessary to conduct studies with a larger sample size and broader scope in order to represent the profile of bladder exstrophy patients more accurately. Furthermore, long-term studies are also important to assess clinical outcomes and factors influencing the anatomical and functional outcomes of bladder exstrophy patients.
